# Molecular basis underlying changes of brain entropy and functional connectivity in major depressive disorders after electroconvulsive therapy

**DOI:** 10.1111/cns.14690

**Published:** 2024-03-26

**Authors:** Xiaohui Yu, Kexuan Chen, Yingzi Ma, Tongjian Bai, Shunli Zhu, Defang Cai, Xing Zhang, Kai Wang, Yanghua Tian, Jiaojian Wang

**Affiliations:** ^1^ State Key Laboratory of Primate Biomedical Research, Institute of Primate Translational Medicine Kunming University of Science and Technology Kunming China; ^2^ Yunnan Key Laboratory of Primate Biomedical Research Kunming China; ^3^ Medical School Kunming University of Science and Technology Kunming China; ^4^ Department of Neurology The First Hospital of Anhui Medical University Hefei China; ^5^ School of Life Science and Technology University of Electronic Science and Technology of China Chengdu China; ^6^ The Second People's Hospital of Yuxi The Affiliated Hospital of Kunming University of Science and Technology Yuxi China; ^7^ Anhui Province Key Laboratory of Cognition and Neuropsychiatric Disorders Hefei China; ^8^ School of Mental Health and Psychological Sciences Anhui Medical University Hefei China; ^9^ Collaborative Innovation Center of Neuropsychiatric Disorders and Mental Health Hefei China; ^10^ Anhui Province Clinical Research Center for Neurological Disease Hefei China; ^11^ Institute of Artificial Intelligence Hefei Comprehensive National Science Center Hefei China

**Keywords:** brain entropy, cerebellar, electroconvulsive therapy, gene expression, major depressive disorder

## Abstract

**Introduction:**

Electroconvulsive therapy (ECT) is widely used for treatment‐resistant depression. However, it is unclear whether/how ECT can be targeted to affect brain regions and circuits in the brain to dynamically regulate mood and cognition.

**Methods:**

This study used brain entropy (BEN) to measure the irregular levels of brain systems in 46 major depressive disorder (MDD) patients before and after ECT treatment. Functional connectivity (FC) was further adopted to reveal changes of functional couplings. Moreover, transcriptomic and neurotransmitter receptor data were used to reveal genetic and molecular basis of the changes of BEN and functional connectivities.

**Results:**

Compared to pretreatment, the BEN in the posterior cerebellar lobe (PCL) significantly decreased and FC between the PCL and the right temporal pole (TP) significantly increased in MDD patients after treatment. Moreover, we found that these changes of BEN and FC were closely associated with genes' expression profiles involved in MAPK signaling pathway, GABAergic synapse, and dopaminergic synapse and were significantly correlated with the receptor/transporter density of 5‐HT, norepinephrine, glutamate, etc.

**Conclusion:**

These findings suggest that loops in the cerebellum and TP are crucial for ECT regulation of mood and cognition, which provides new evidence for the antidepressant effects of ECT and the potential molecular mechanism leading to cognitive impairment.

## Graphical abstract

We explored the molecular basis underlying changes of brain entropy and functional connectivity in individuals with major depression disorder after electroconvulsive therapy using resting‐state functional magnetic resonance imaging, neurotransmitter density maps, and transcriptomic data.

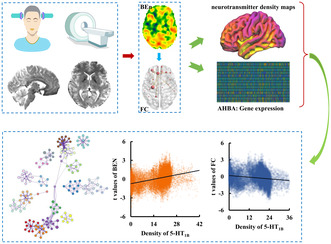

## 1 INTRODUCTION

Electroconvulsive therapy (ECT) has been demonstrated to be a rapid and effective clinical treatment for severe refractory depression, especially in patients with drug resistance.^1^ A large number of recent studies have demonstrated that ECT can reverse neuroplasticity deficits in patients with major depressive disorder (MDD) and improve depressive symptoms by reshaping the structures and functions of specific brain regions or normalizing/reorganizing functional interactions of specific circuits.^2^, ^3^, ^4^, ^5^, ^6^ Given that systematic orderliness and intrinsic balance are critical to maintain normal brain functional loops, thus, quantitatively characterizing global or systematic functional regularity, complexity, and dynamics may better reveal neural mechanism of ECT.

Entropy is a fundamental concept derived from physics and information theory and measures the complexity, randomness, and predictability of dynamic processes.^7^, ^8^, ^9^ High entropy implies high disorder or high irregularity of systematical activity and also indicates a greater capacity to process information.^10^ Brain entropy (BEN) has been reliably mapped in normal brains^11^ and brain disorders including Alzheimer's disease,^12^, ^13^ attention deficit hyperactivity disorder,^8^ schizophrenia,^14^, ^15^ bipolar disorder,^16^ and depression.^17^ A previous study reported that BEN can provide some unique information that the traditional methods such as cerebral blood flow and fractional amplitude of low‐frequency fluctuation cannot identify.^18^ In a word, BEN is a reliable and sensitive method, which shows great potential to quantitatively characterize brain status in normal and disease states.

Recent advances in the field of neuroimaging‐transcriptomic spatial association analyses, marked by the combination of brain imaging data and whole‐brain gene expression atlases, provide a viable way to reveal the molecular basis for structural and functional changes in brain diseases.^19^, ^20^, ^21^ The public availability of the Allen Human Brain Atlas (AHBA)^22^ has briged the gap to link the transcriptomic expression profiles to neuroimaging phenotypes. In addition to transcriptomic regulator, the neurotransmitters such as serotonin, gamma‐aminobutyric acid (GABA), and dopamine, which are closely related to mood regulation have been demonstrated to play a key role in the onset and development of MDD.^23^, ^24^ A recent study constructed a whole‐brain three‐dimensional normative atlas of 19 receptors and transporters across nine different neurotransmitter systems using positron emission tomography data from more than 1200 healthy individuals.^25^ The transcriptomic and neurotransmitter datasets provide excellent tools to explore the therapeutic molecular mechanisms underlying the antidepressant effect of ECT.

The goal of this study was to investigate the genetic and molecular mechanism of ECT therapy for MDD patients using resting‐state functional MRI (rs‐fMRI), transcriptomic and neurotransmitter receptor data. The rs‐fMRI data from 46 MDD patients before and after ECT and 46 healthy controls (HCs) were acquired. BEN and FC were calculated for each subject and compared to reveal the changes of brain status and functional couplings in MDD patients after ECT to determine treatment efficacy. Then, transcriptomic and neurotransmitter receptor datasets were used to identify the molecular basis of ECT. Finally, functional enrichment and protein–protein interactions (PPIs) analyses of the significantly associated genes were performed to determine the functional characteristics of the associated genes.

## 2 MATERIALS AND METHODS

### 2.1 Subjects

A total of 92 right‐handed subjects including 46 patients with MDD (9 males/37 females, age 39.91 ± 12.25 years) and 46 age‐ and sex‐matched HCs (11 males/35 females, age 35.48 ± 11.77 years) were included in this study. Patients with substance abuse or dependence, severe physical illness, neurological disorders, pregnancy, comorbid psychiatric disorders, and contraindications to MRI were excluded. MDD patients received antidepressant medication as usual during ECT to maintain treatment stability to exclude medication effects. The 17‐item Hamilton Depression Scale (HAMD) was used to assess the severity of depression symptoms of MDD patients.^26^ Patients completed the scale 12–24 h before the first ECT treatment and 24–72 h after the last ECT treatment. All participants signed written informed consent, and the study was approved by the local Ethics Committee of Anhui Medical University.

### 2.2 ECT procedures

This study used the Thymatron System IV integrated ECT system at Anhui Provincial Mental Health Center (Somatics, Lake Bluff, IL, USA) with a modified bifrontal ECT protocol. ECT was performed three times a week, one day apart, with a weekend break. When ECT was given, all MDD patients were anesthetized with isoproterenol and paralyzed with succinylcholine and atropine to relax muscle tissue. Seizure activity was monitored with EEG. During treatment, the initial energy percentage was set according to the age of each participant (e.g., 50% for 50‐year‐old patients), and the stimulation intensity was uniformly adjusted in 5% increments of the maximum charge (~1000 milli‐Coulomb) until a seizure was detected. The details for ECT procedures are provided in our previous studies.^5^, ^6^, ^27^

### 2.3 Resting‐state fMRI data acquisition and preprocessing

The rs‐fMRI data were collected using 3.0T GE MRI scanner (Discovery GE750w). All MDD patients underwent two rs‐fMRI scans 12–24 h before the first ECT and 24–72 h after the last ECT. HCs were also scanned to determine pretreatment structural and functional changes in the patients. All participants were asked to close their eyes, relax, stay awake, and not to think of anything in particular during the scans. The rs‐fMRI images were acquired using a standardized echo planar imaging (EPI) sequence with the same parameters: repetition time/echo time ratio = 2400/30 ms, flip angle = 90°, matrix size = 64 × 64, field of view = 192 × 192 mm^2^, 46 slices, voxel size = 3 × 3 × 3 mm^3^, and 217 volumes.

The preprocessing of rs‐fMRI data mainly comprised the following steps: discarding the first 10 volumes, realign, normalizing to the Montreal Neurological Institute (MNI) template, resampled to 3 × 3 × 3 mm^3^, smoothing with a Gaussian kernel of 8‐mm full‐width at half‐maximum, filtering with temporal band path (0.01–0.1 Hz), regressing out Friston‐24 motion parameters, white matter, global mean, and cerebrospinal fluid signals. In addition, to exclude the head motion effects, scrubbing method with cubic spline interpolation was used to eliminate bad volume with mean frame‐wise displacement (FD) > 0.2 mm.

### 2.4 BEN mapping

After preprocessing, BEN mapping was performed for each subject. BEN was calculated at each voxel using fuzzy entropy (FuzzyEn).^28^ Similar to the existing measures of approximate entropy^29^ and sample entropy (Entropy),^30^ FuzzyEn is the negative natural logarithm of the conditional probability that two vectors similar for the *M* points remain similar for the next *M* + 1 points. The match is defined by a cutoff threshold *r*, and the optimal window length “*M*” was set to 2 and the optimal cutoff threshold *r* was set to 0.2. A two‐sample *t*‐test was used to determine the difference in BEN between patients before treatment and healthy individuals, and a paired *t*‐test was used to determine the changes of BEN in MDD patients before and after ECT treatment (post – pre). The significant level was set *p* < 0.05 corrected using a cluster‐level Monte Carlo simulation method (cluster‐forming threshold at voxel‐level *p* < 0.001).

### 2.5 Changes of FC after ECT

After statistical analysis, the regions with significant differences in BEN were defined as seed regions for FC analysis to explore the changes of functional couplings in MDD patients after ECT. FC was calculated between the average time series of each seed region and the time series of each voxel in the whole brain. To identify ECT effects on functional interaction, paired *t*‐test was used to determine longitudinal changes of FC in MDD patients before and after ECT (post – pre). Two‐sample *t*‐test was used to determine differences in FC between HCs and MDD patients. The significant level was set at *p* < 0.05 corrected using a cluster‐level Monte Carlo simulation method (cluster‐forming threshold at voxel‐level *p* < 0.001).

Meanwhile, to reveal whether the brain areas showing changes of BEN and FC were associated with clinical characteristics of HAMD scores assessing the severity of depressive symptoms and MMSE scores assessing cognitive and memory functioning, correlation analyses between changes, percentage of changes of BEN or FC before and after therapy and changes, percentage of changes of clinical variables before and after therapy were executed, respectively. The significant level was set at *p* < 0.05 with family‐wise discovery rate (FDR) corrected.

### 2.6 Transcription‐neuroimaging association analysis

To explore the genetic basis for ECT treatment response, we evaluated the relationship between gene expression profiles and changes of BEN, FC in MDD patients before and after ECT to identify the genes significantly associated with the treatment efficacy of ECT. The brain gene expression data were provided by the AHBA dataset which was derived from the brain samples of six human donors (5 males and 1 female) (http://human.brain‐map.org/). The tissue samples from 3702 spatially distinct locations were preprocessed using developed pipeline by Arnatkeviciute et al.^31^ and finally yielded 10,027 normalized gene expression data. We projected the *t*‐maps of the statistical analyses onto the brain atlas constructed by Schaefer et al.^32^ which divided the left and right hemispheres into 500 subregions. The mean *t* value for each subregion in the left hemisphere was extracted. Spatial Pearson correlation analysis was performed between gene expression data and mean *t* values. Multiple comparisons were corrected using the FDR‐BH method with *p* < 0.05.

### 2.7 Gene enrichment analysis

Gene ontology (GO) biological processes and Kyoto Encyclopedia of Genes and Genomes (KEGG) pathway analysis of significantly associated genes were performed using Metascape (https://metascape.org/). Overlapping genes were performed in the same way. For the aforementioned enrichment analyses, items with *p* < 0.01, a minimum overlap value of 3, and a minimum enrichment value of 1.5 (enrichment factor is the ratio between observed counts and expected counts by chance) were collected and grouped into clusters based on the similarity of their affiliation. For hierarchical clustering of enriched terms, Kappa scores were used as a similarity measure and subtrees with a similarity >0.3 were considered as a cluster. The most statistically significant term in a cluster was chosen to represent that cluster.

### 2.8 Protein–protein interaction analysis

PPI analysis was carried out using STRING v11.0 (https://cn.string‐db.org/) to determine whether the genes significantly associated with ECT efficacy (|*r*| ≥ 0.15) could construct a PPI network with a highest confidence interaction score of 0.9. Gene with degree values (i.e., the number of edges connected to a gene) more than the mean plus one standard deviation were defined as hub genes. The overlapping genes also performed the above operations. Whole‐brain gene expression levels of the top 5 hub genes rankings by node degree were analyzed using the Neurosynth toolkit (https://neurosynth.org/). Afterward, the brain was divided into 116 subregions using automated anatomical labeling (AAL) atlases,^33^ and the average gene expression level was calculated for each sub‐region. The gene expression levels of the top ten ranked subregions are displayed. And correlation analysis was used to reveal the association between brain BEN and FC changes and hub gene expression. Using the brain atlas constructed by Schaefer et al.,^32^ the *t*‐map (post‐pre) and the whole‐brain gene expression profiles of the overlap genes were divided into 1000 ROIs and correlation analysis between the 1000 *t* values and 1000 expression values were performed. The significance level was set at *p* < 0.05 and corrected using Bonferroni.

### 2.9 Spatial correlation with neurotransmitter density

Neurotransmitter density maps were derived from PET images of more than 1200 healthy individuals and included 19 different neurotransmitter receptors and transporters for nine different neurotransmitter systems (dopamine, norepinephrine, serotonin, acetylcholine, glutamate, GABA, histamine, cannabinoid, and opioid). All neurotransmitter receptor density maps were downloaded from this linkage (https://github.com/netneurolab/hansen_receptors/tree/main/data/PET_nifti_images).^25^ To identify the molecular basis for ECT, spatial Spearman rank correlations between neurotransmitter density maps and *t*‐statistic maps of BEN and FC (post‐pre) were performed. The significance was determined using 5000 permutation tests. For permutation tests, BEN or FC map of MDD patient before and after ECT was mixed and was randomly divided into two groups and each group has 46 BEN or FC maps. Then, paired *t*‐tests were performed to identify the changes of BEN or FC and Spearman rank correlations between this statistical *t* map and neurotransmitter density maps were performed. This procedure was repeated 5000 times, and a distribution for the spatial correlation values was obtained. Finally, the location of the true spatial correlation value in the distribution was calculated and was defined as the statistical *p* value. The significance level was set at *p* < 0.05 and corrected using Bonferroni.

## 3 RESULTS

### 3.1 Demographic and clinical characteristics

Table 1 summarizes the demographic and clinical characteristics of all participants. For patients with MDD after ECT, a significant reduction in depressive symptoms was observed (*p* = 3.44 × 10^−9^). Patients with MDD had significantly higher depressive load before and after ECT treatment compared to HCs (MDD before ECT vs. HCs: *p* = 1.47 × 10^−16^; MDD after ECT vs. HCs: *p* = 5.57 × 10^−7^). MMSE scores were significantly lower in MDD patients before and after ECT compared to HCs (MDD vs. HCs: *p* = 1.02 × 10^−5^ and MDD after ECT vs. HCs: *p* = 2.28 × 10^−7^).

**TABLE 1:** Demographic and clinical information.

Variables	MDD		HCs	*p*‐value
Number of subjects	46		46	
Gender (male/female)	9/37		12/34	0.46
Age (years: mean ± SD)	39.91 ± 12.25		35.00 ± 11.91	0.059
Education (years)	10.15 ± 4.54		13.67 ± 3.78	1.05 × 10^−04^
HAMD scores (mean ± SD)	ECT before	ECT after	1.83 ± 1.97	
	23.85 ± 5.98	6.74 ± 5.57		
MMSE scores (mean ± SD)	ECT before	ECT after	29.45 ± 1.22	
	28.38 ± 1.89	28.08 ± 0.71		
Age of onset (years)	35.50 ± 11.45			
Duration of illness (months)	55.98 ± 68.36			
Medication (*n* patients)	46			

*Note*: Age and years of education were compared using a Mann–Whitney *U* test. Comparison of gender was carried out using chi‐square test. Mann–Whitney *U* test were used for HAMD and MMSE comparisons before and after ECT. Checking the normal distribution of quantitative data using the Kolmogorov–Smirnov–Lilliefors method.

Abbreviations: HAMD, Hamilton depression scale; HCs, healthy controls; MDD, major depressive disorder; MMSE, Mini‐Mental State Examination.

### 3.2 Changes in BEN after ECT treatment

**FIGURE 1:**
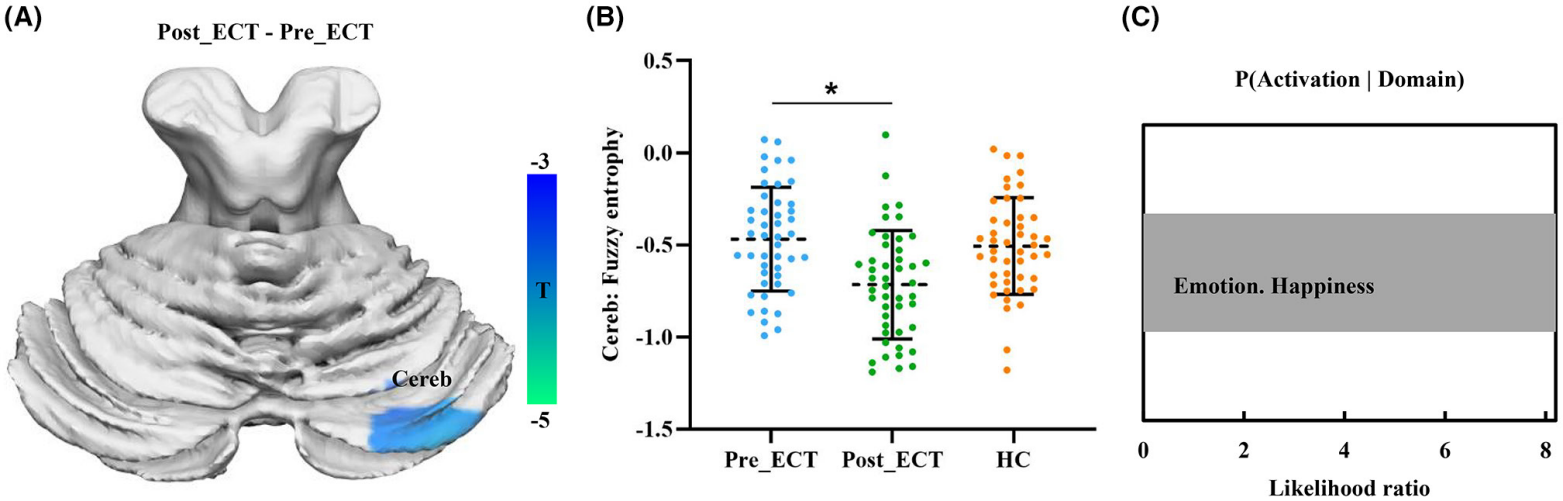
Changes of brain entropy (BEN) in MDD patients after ECT. (A) Patients with MDD showed significantly decreased BEN in posterior cerebellar lobule (Cereb) after ECT. (B) MDD patients before and after ECT showed no difference in BEN compared to healthy controls. (C) Functional decoding of posterior cerebellar lobule found that this area is mainly involved in emotion, especially happiness processing.

To investigate the changes of BEN after ECT in patients with MDD, we used paired *t*‐test and found that BEN in the right posterior cerebellum lobule (PCL) of MDD patients decreased significantly after ECT (Figure 1A). All three sets of data were found to conform to a normal distribution. There was no significant difference in BEN between MDD patients before and after ECT compared to HCs (Figure 1B). Functional decoding of this area using online Neurosynth toolkit found that PCL was mainly associated with emotion, especially happiness processing (Figure 1C).

### 3.3 Changes in FC of PCL after ECT treatment

**FIGURE 2:**
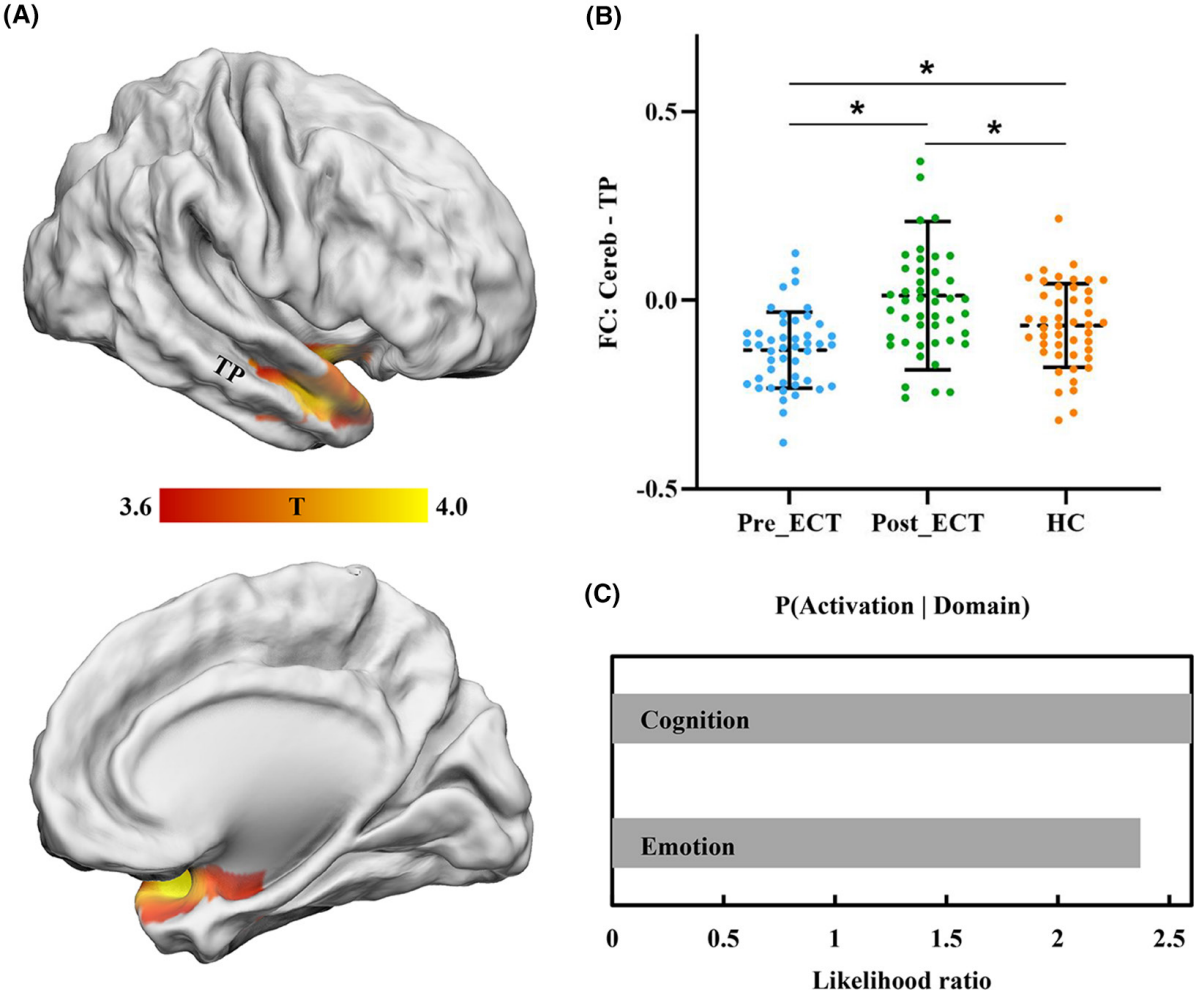
Functional connectivity (FC) difference with posterior cerebellar lobule (Cereb) in MDD patients after ECT. (A) Significantly increased FC between posterior cerebellar lobule and right temporal pole (TP) was found in MDD patients after ECT. (B) Compared to healthy controls, FC between Cereb and TP decreased in MDD patients and increased after ECT. (C) Functional decoding found Cereb and TP mainly involved in emotion and cognition functions.

We also analyzed the effect of ECT on functional coupling of the right PCL. We found that the FC between PCL and right temporal pole (TP) significantly increased in MDD patients after ECT compared to before treatment (Figure 2A). The group after ECT was found not to conform to a normal distribution, and the Mann–Whitney *U* test was applied to this group. MDD patients before treatment also showed significantly decreased FC between PCL and right TP compared to HCs suggesting ECT could normalize abnormal functional coupling in MDD patients (Figure 2B). Using online Neurosynth toolkit, we found that this area is mainly involved in emotion and cognition processing (Figure 2C). Moreover, the change in FC between PCL and right TP before and after treatment was significantly negatively correlated with changes of MMSE scores (uncorrected, Figure S1).

### 3.4 Gene enrichment and PPI analyses

**FIGURE 3:**
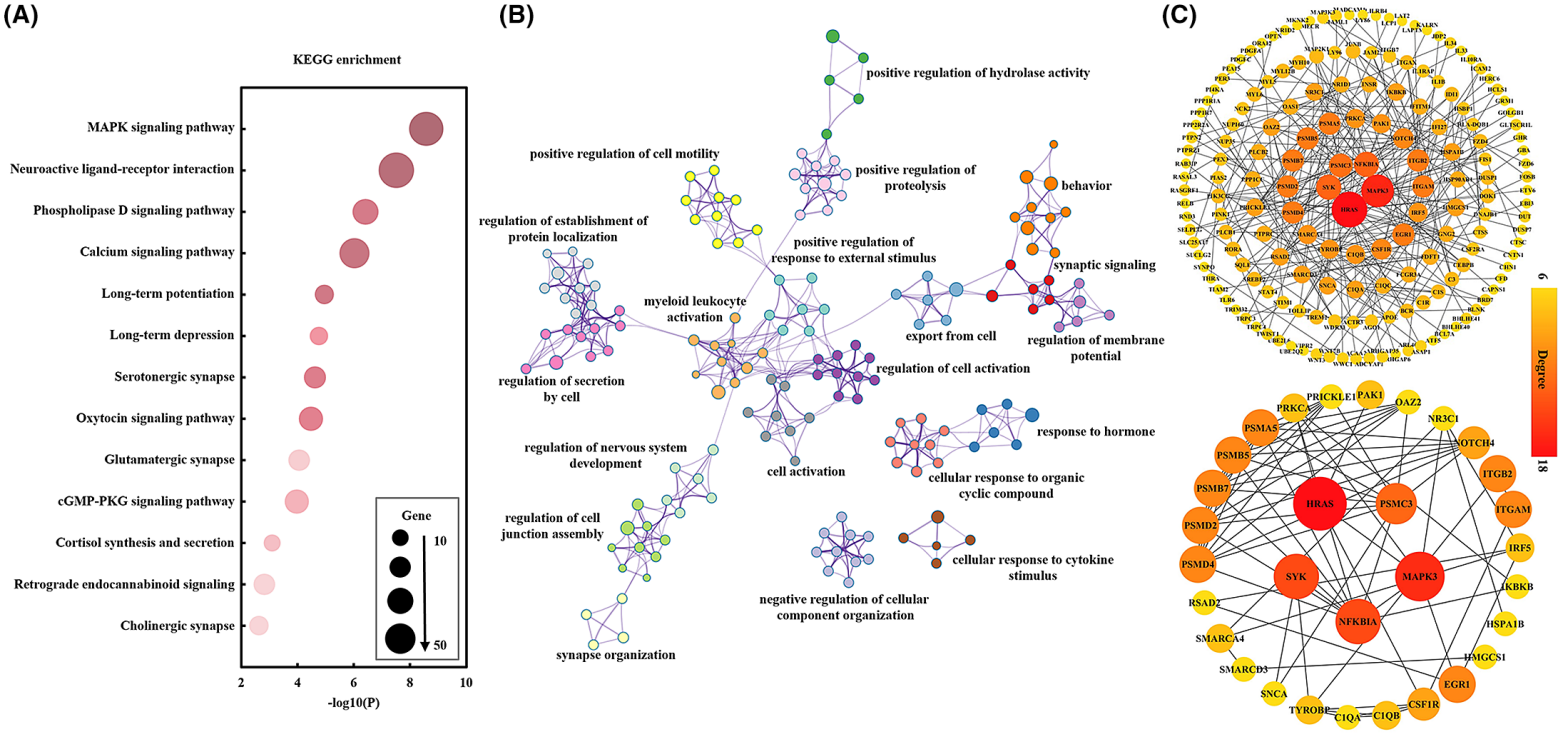
Functional enrichment and PPI analysis of the genes associated with changes of BEN after ECT. (A) The signal pathway enrichment results of KEGG for the associated genes. (B) GO‐enriched terms networks colored by cluster identity, where nodes with the same cluster identity are usually close to each other. (C) PPI network (above) and hub genes network (bottom).

**FIGURE 4:**
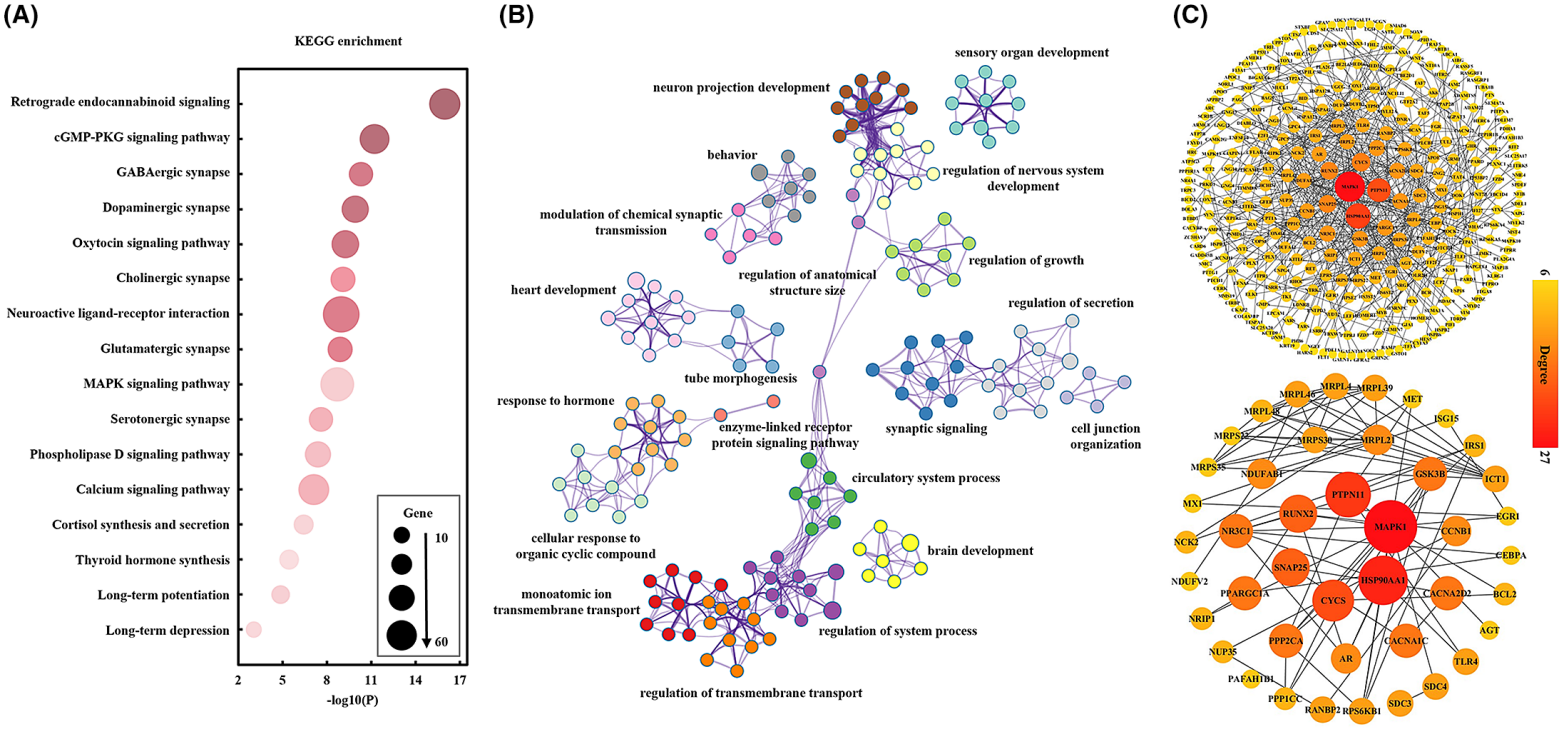
Functional enrichment and PPI analysis of the genes associated with changes of FC after ECT. (A) KEGG signal pathway enrichment results. (B) GO‐enriched terms networks colored by cluster identity, where nodes with the same cluster identity are usually close to each other. (C) PPI network (above) and hub genes network (bottom).

To explore the molecular mechanisms underlying the changes in BEN and FC after ECT, we performed spatial Pearson's correlation analysis of statistical differences in BEN or FC with AHBA profiles to identify the associated genes. Enrichment analysis of these genes revealed that the KEGG pathways were involved in neurotransmitter release (cholinergic synapse, clutamatergic synapse, and serotonergic synapse), in vivo hormone synthesis and release (cortisol synthesis and secretion, thyroid hormone synthesis), signal transduction (retrograde endocannabinoid signaling, cGMP‐PKG signaling pathway, oxytocin signaling pathway, neuroactive ligand‐receptor interaction, MAPK signaling pathway, phospholipase D signaling pathway, and calcium signaling pathway), and synaptic plasticity (long‐term potentiation and long‐term depression). We also enriched nine of these pathways in overlapping gene (Figure S2). In addition, three pathways, GABAergic synapse, dopaminergic synapse, and thyroid hormone synthesis, were specific to FC and contributed only to the functional connectivity (FC) changes in the brain after ECT treatment. (Figures 3A and 4A). The biological processes including synaptic signaling, modulation of chemical synaptic transmission, response to hormone, positive regulation of hydrolase activity, regulation of cell activation, monatomic ion transmembrane transport, regulatory systems, and brain development were found to be significantly associated with these genes (Figures 3B and 4B). PPI network analysis identified the hub genes that may play the key roles in regulation of ECT efficacy (Figures 3C and 4C).

### 3.5 Whole‐brain expression levels of the Hub genes

**FIGURE 5:**
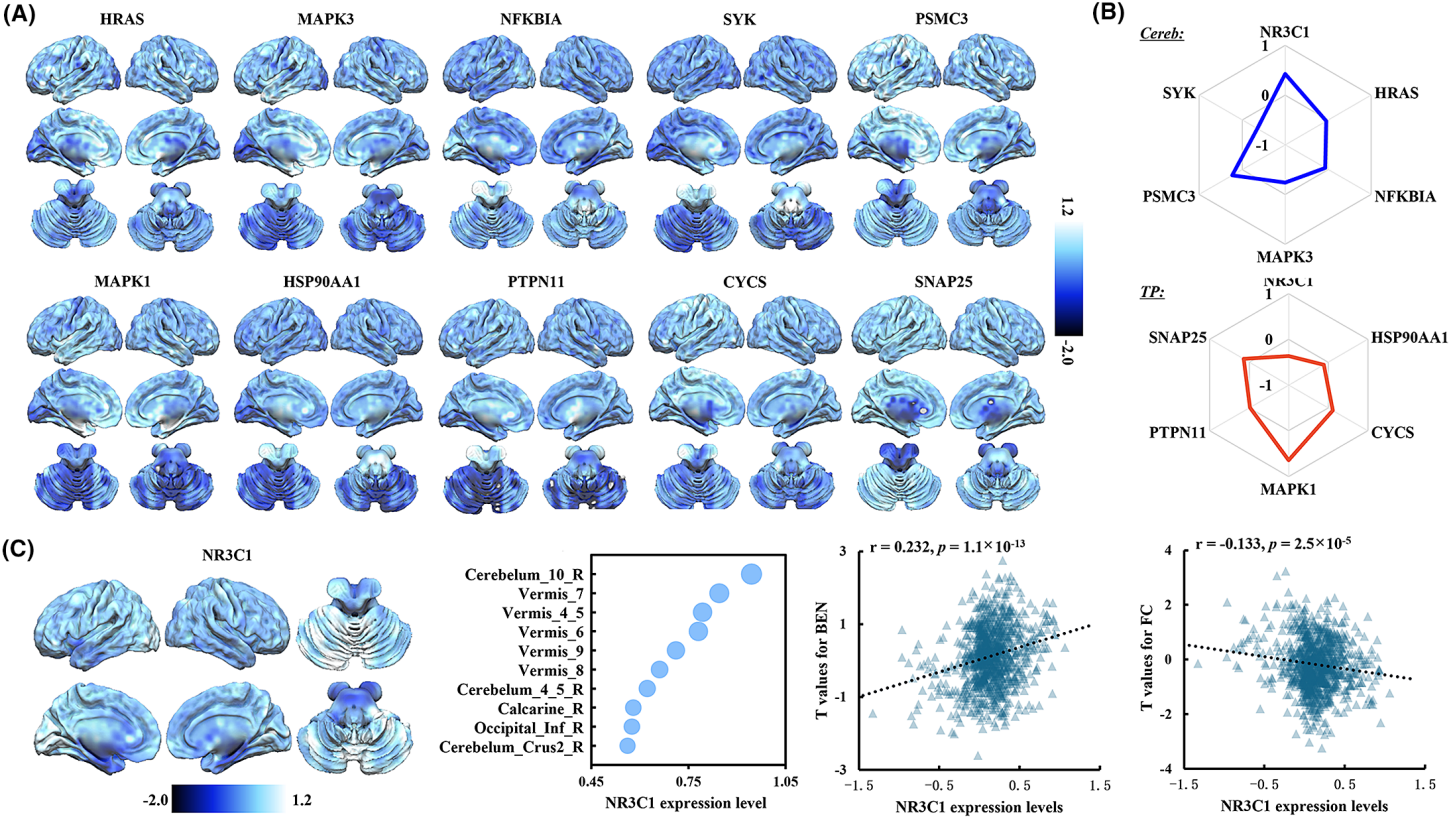
Whole‐brain expression levels of the overlap hub genes. (A) Whole‐brain expression levels of the top 5 ranked genes identified using BEN (up panel) and FC (bottom panel). (B) Average expression levels of the top 5 hub genes and 1 overlap hub gene identified by both BEN and FC for brain regions showing difference in BEN (PCL: top panel) or FC (TP: bottom panel). (C) The brain‐wide gene expression of the overlapped gene *NR3C1* and the top 10 brain areas showing highest expression level were shown. Significant spatial correlations of the expression of *NR3C1* gene with the changes of BEN and FC were found (*p* < 0.05, Bonferroni corrected).

The whole brain expression profiles of the top 5 hub genes identified using BEN and FC were exhibited in Figure 5A, respectively. In all the top 10 hub genes, the genes of *HRAS*, *NFKBIA*, *PSMC3*, *SYK* and *PTPN11* had the highest expression level in the basal ganglia (caudate nucleus, pallidum and putamen); the genes of *MAPK1*, *MAPK3*, and *SNAP25* had the highest expression in the amygdala and the genes of *HSP90AA1* and *CYCS* showed the highest expression in cerebellum or/and the vermis (Figure S3). In the PCL showing BEN difference in MDD patients after ECT, the gene of *NR3C1* showed high positive expression level while the gene of *SYK* showed high negative expression level. In the right TP showing changed FC, the gene of *MAPK1* showed high positive expression level while the gene of *NR3C1* showed high negative expression level (Figure 5B). For all the hub genes, the gene of *NR3C1* was identified by both BEN and FC. The expression level of *NR3C1* is not only highly expressed in cerebellum and vermis but also in inferior occipital gyrus. Moreover, the expression level of *NR3C1* was positively correlated with the changes of BEN while negatively correlated with the changes of FC in MDD patients before and after treatment (Figure 5C).

### 3.6 Association with neurotransmitter density maps

**FIGURE 6:**
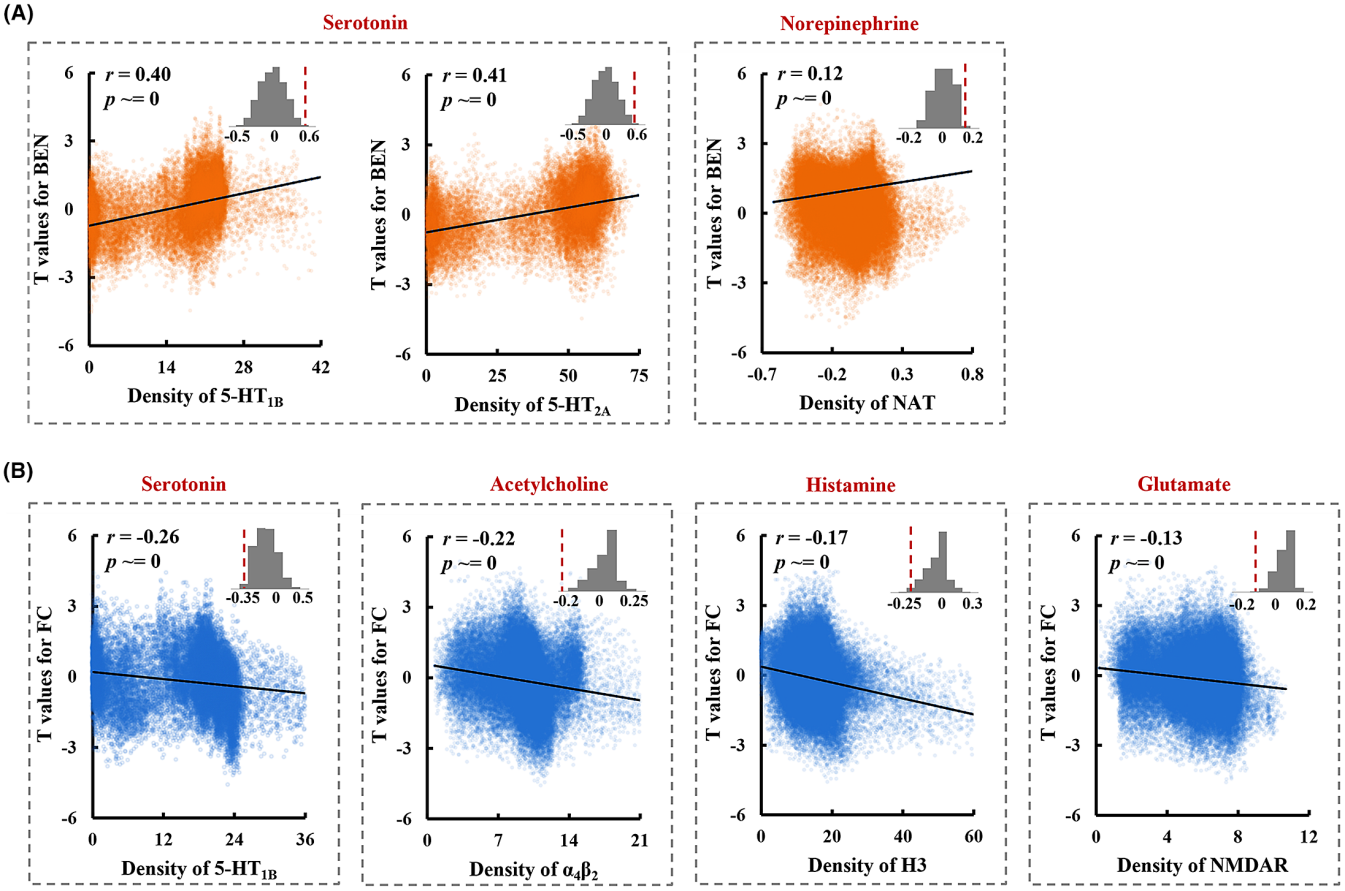
Association with neurotransmitter density maps. (A) Neurotransmitter receptors density maps of serotonin (5‐HT_1B_, 5‐HT_2A_) and norepinephrine (NAT) were significantly positively correlated with *t*‐map of BEN changes after ECT (*p* < 0.05, Bonferroni corrected). (B) Significant negative correlation between neurotransmitter receptors density maps of serotonin (5‐HT_1B_), acetylcholine (α_4_β_2_), histamine (H3), glutamate (NMDAR) and *t*‐map of changes in FC after ECT (*p* < 0.05, Bonferroni corrected).

To identify molecular basis for ECT, spatial correlation between changes of BEN or FC and neurotransmitter density maps were performed. We found that the changes of BEN in MDD patients after ECT compared to before treatment were positively correlated with the receptor/transporter densities of serotonin (5‐HT_1B_: *r* = 0.4; 5‐HT_2A_: *r* = 0.41) and norepinephrine (NAT: *r* = 0.12) (Figure 6A). In addition, we found that the changes of FC in MDD patients after ECT compared to before treatment were negatively correlated with the density of the receptors/transporters of serotonin (5‐HT_1B_: *r* = −0.26), acetylcholine (α_4_β_2_: *r* = −0.22), histamine (H3: *r* = −0.17) and glutamate (NMDAR: *r* = −0.13) (Figure 6B).

## 4 DISCUSSION

This study combined BEN, FC, transcriptome and neurotransmitter receptor density maps to reveal the neural and molecular mechanisms for ECT. We found that ECT effects on depression relief and cognitive impairment may be regulated by cerebellum‐temporal pole (PCL‐TP) circuit. Moreover, we found that the treatment outputs of ECT were closely associated with neurotransmitter density of serotonin, norepinephrine, acetylcholine, histamine, and glutamate and genes' expression level, especially the expression level of the *NR3C1* gene which was found to be significantly correlated with the changes in both BEN and FC before and after treatment. Our findings revealed an important role of PCL‐TP circuit in regulating emotion and cognition as well as its corresponding molecular basis in MDD patients receiving ECT.

In recent years, more and more researches about cerebellar structure and function were performed.^34^ It has been found that the cerebellum not only regulates somatic movements and balance, but also plays a dominant role in cognition and emotion as part of the dysfunctional brain circuitry in depression.^35^, ^36^ The posterior lobe of the cerebellum is a key part of the brain that regulates emotion and cognition,^37^ and posterior lobe lesions can even lead to cerebellar cognitive‐emotional syndrome.^38^, ^39^ The reduced BEN in the PCL suggests that MDD patients have reduced irregularity and increased orderliness of brain activity in the PCL after ECT treatment, which may account for depression relief while cognitive impairment observed in MDD patients after ECT. ECT enhances the orderliness of the PCL and may help to maintain homeostasis in other brain regions. This compensatory mechanism is supported by the extensive cerebellar‐brain connectivity analysis. The previous studies found that extensive cerebellar‐cortex interactions underlie the cerebellum's regulation of movement, thought, and emotion.^40^, ^41^ Numerous studies have demonstrated that disruption of cerebellar‐cortex FC is one of the neurobiological changes in depression^42^, ^43^ and this abnormal cerebellar‐cortex FC can be ameliorated by ECT.^44^, ^45^ These findings collectively demonstrate that altered cerebellar‐brain resting‐state FC is strongly associated with ECT‐induced antidepressant effects, which is consistent with our findings that we found that increased FC between PCL and TP in MDD patients after ECT. The TP plays an important role in emotional processing, and TP lesions lead to emotional deficits.^46^, ^47^ However, we found that increased FC between PCL and TP was negatively correlated with MMSE scores, which indicated that ECT induced cognition and memory declines coming with enhanced FC between PCL and TP. Short‐term cognitive impairment after ECT has been confirmed by a large number of studies but was not evident in our scale results.^48^, ^49^, ^50^ Therefore, we concluded that PCL‐TP circuit is important for both cognition and emotion and the dynamic balance is crucial to maintain brain normal functions.

We found that the therapeutic effect of ECT involves a variety of neurobiological mechanisms. In addition to the changes of macroscopic brain circuits, we also observed a diverse range of molecular pathway changes associated with ECT. ECT is significantly associated with the expression of genes which can promote neurogenesis and synaptic plasticity by regulating diverse signaling pathways and affecting the activity of multiple neurotransmitters in the central nervous system to induce structural and functional changes in the brain for depressive symptoms relief. The monoamine theory, one of the early proposed biological mechanisms of MDD, involves serotonin, norepinephrine, and dopamine (5‐HT, DE, and DA). Monoamine neurotransmitters are the primary neural basis of emotion and are associated with punishment (disgust or sadness), fear (anger), and reward (pleasure).^51^ The deficiencies in monoamine levels were found to be prevalent in patients with MDD.^52^ Some findings suggest that ECT could down‐regulate 5‐HT_2_ receptors in the brains of MDD patients.^53^ Our results also confirm that the antidepressant effects of ECT are related to the serotonergic synapse pathway suggesting that serotonin is essential for the ordering of brain systems and FC between brains. However, follow‐up experiments are needed to further reveal the influence of the serotonergic system in the action of ECT.

Several studies have reported the structural and functional defects of excitatory glutamatergic neurons as well as inhibitory GABA interneurons in patients with MDD leading to signaling deficits in cortical and hippocampal regions and resulting in reduced responses to emotional stimuli.^54^, ^55^, ^56^ However, the effects of ECT on the glutamatergic system are unclear and controversial. Some researchers have reported that ECT increases glutamatergic levels in the subgenual anterior cingulate cortex (sgACC) and decreases glutamatergic levels in left hippocampus in depressed patients.^57^ Our results confirm that glutamate after ECT plays an important role in modulating FC between brain regions. This process involves pathways such as retrograde endocannabinoid signaling, calcium signaling pathway, and long‐term depression. Endogenous cannabinoids are the most classical retrograde messengers at synapses in various brain regions. They are released from postsynaptic neurons during postsynaptic depolarization and/or during receptor activation, activate CB1 receptors (CB1R) in presynaptic terminals, and inhibit the release of the inhibitory transmitter GABA or the excitatory transmitter glutamate by inhibiting Ca^2+^ channels. Endogenous cannabinoids cause short‐term synaptic plasticity or mediate long‐term synaptic changes.^58^ ECT may improve mood disorders in depressed patients by modulating endogenous cannabinoids, mediating synaptic plasticity, and normalizing the glutamatergic system.^59^ In addition, histamine and histamine receptor H3 are expressed in the central nervous system and activate the MAPK pathway to regulate behaviors including anxiety and cognition. H3 receptors can also influence the balance of different neurotransmitters by regulating the release of other neurotransmitters (serotonin and dopamine etc.).^60^ Therefore, MDD is not caused by dysfunction of a single neurotransmitter and its receptor/transporter, but by the abnormalities of multiple neurotransmitters and abnormal interactions with each other. Therefore, the therapeutic mechanism of ECT for MDD may be through modulating the patient's mood and cognition by regulating multiple neurotransmitters.

To further identify the key genes regulating the therapeutic effects of ECT, the hub genes were also analyzed and were found to mainly play important roles in biological processes such as immune response, inflammation, neurotransmitter signaling, and neuroplasticity. For example, the gene of *PTPN11* is essential for the activation of RAS‐MAPA signaling and is mainly involved in synaptic plasticity and memory formation.^61^ The gene of *SNAP25* plays an important role in triggering synaptic vesicle fusion and regulating the release of presynaptic neurotransmitters.^62^ Its differential expression levels are significantly associated with a variety of psychiatric disorders, including MDD.^63^, ^64^ The hub gene of *NR3C1* found by both BEN and FC encodes the glucocorticoid receptor (GR), an important component in the hypothalamic–pituitary–adrenal (HPA) axis. In patients with MDD, there is a significant increase in the methylation level of the *NR3C1* promoter region, resulting in reduced expression levels of this gene and abnormal GR signaling.^65^ The reduced expression of *NR3C1* and abnormal GR signaling result in the compromised negative feedback regulation of the HPA axis^66^ making cortisol levels at a high level^52^, ^67^, ^68^ to impair cognitive function in MDD patients.^69^ Although it is unclear and uncertain how changes in hormone levels in the HPA axis affect the improvement of MDD symptoms, ECT has been shown to reduce cortisol‐induced inhibition of neuroplasticity in animal experiments,^70^, ^71^ and our results demonstrate that the *NR3C1* gene may be key to this process.

There are some limitations to declare in our study. First, the AHBA transcriptome data were available for only 6 donors, a small sample size, gender imbalance (1 female and 5 males), and 4 of the donors had only left hemisphere brain samples, which may lead to some bias in our statistical analysis. Second, our sample size was moderate, and a larger cohort study is needed to demonstrate the findings in future studies. Third, the transcriptomic and neurotransmitter data are not from the used subjects, the individual differences cannot be excluded.

## 5 CONCLUSION

In this study, we evaluated the therapeutic mechanisms of ECT in patients with MDD using rs‐fMRI. We found that ECT treatment reduced BEN in the PCL and enhanced functional coupling between the PCL and the right TP. Changes in dynamic brain activity after ECT are closely related to the expression levels of genes such as *PTPN11*, *SNAP25*, and *NR3C1* and the density of neurotransmitters such as serotonin, norepinephrine and dopamine. The therapeutic effects of ECT may result from normalizing signaling pathway conduction, neurotransmitter release, and synaptic plasticity to ameliorate depressive symptoms. These findings highlight the important role of the dynamic balance of PCL and its involved circuit of PCL‐TP in regulation emotion and cognition in MDD by ECT.

## CONFLICT OF INTEREST STATEMENT

None.

## Supporting information


Figure S1

## Data Availability

Research data are not shared.
