# Supplementary material for: Molecular basis underlying changes of brain entropy and functional connectivity in major depressive disorders after electroconvulsive therapy
Source: CNS Neurosci Ther. 2024 Mar 26;30(3):e14690. doi: 10.1111/cns.14690 (PMC10964037; doi:10.1111/cns.14690)

**Supplementary Materials**

**Figure S1.** Correlations analyses between behavioral scores and differences in functional indicators in MDD patients after ECT. (A), Correlation of differences in MMSE and HAMD scores with changes in BEN before and after ECT (Left two pictures: pre-post, right two pictures: (pre-post)/pre). (B), Correlation of differences in MMSE and HAMD scores with changes in FC before and after ECT (Left two pictures: pre-post, right two pictures: (pre-post)/pre).


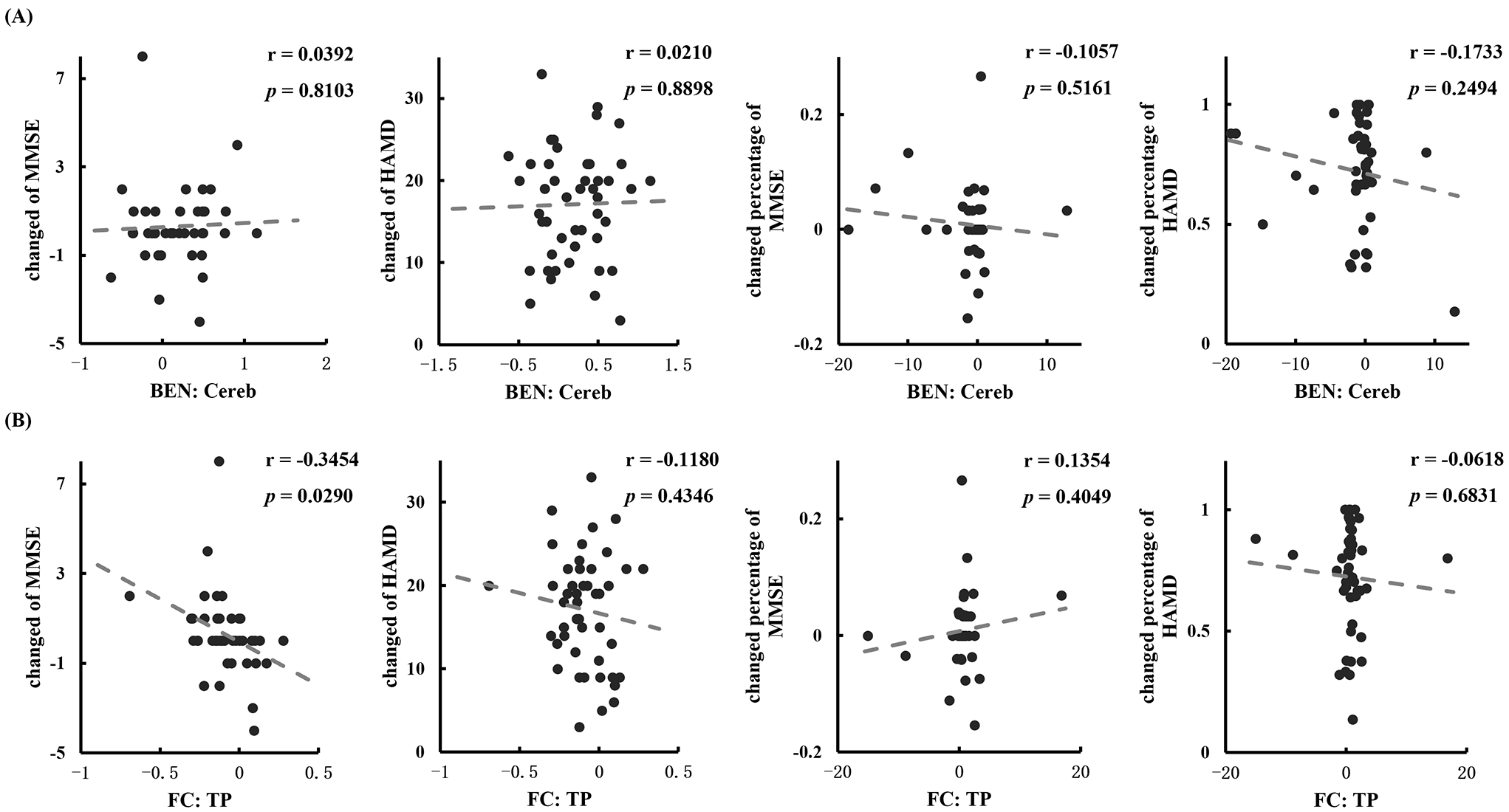


**Figure S2.** Functional enrichment and PPI analysis of overlapping genes associated with changes of BEN and FC after ECT. (A) The signal pathway enrichment results of KEGG for overlapping genes. (B) GO-enriched terms networks colored by cluster identity, where nodes with the same cluster identity are usually close to each other. (C) PPI network (above) and hub genes network (bottom).


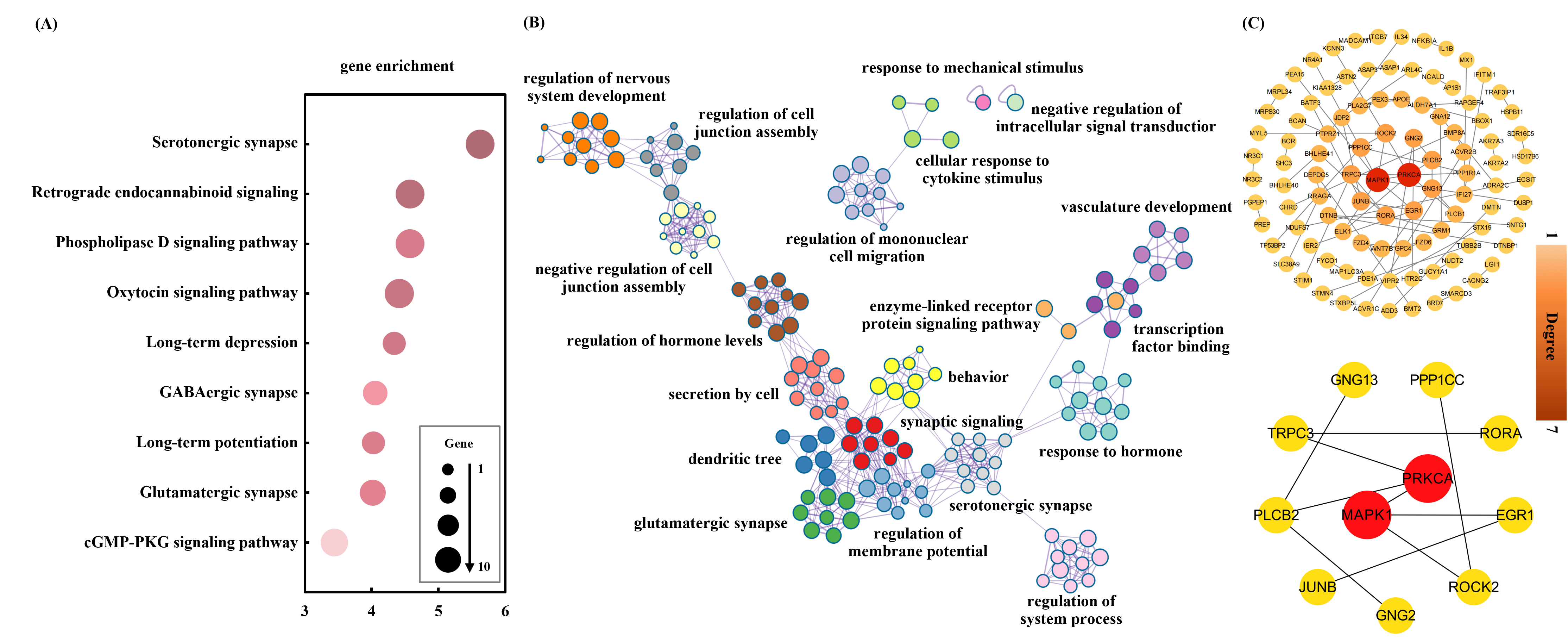


**Figure S3.** The top 10 regions of the brain with the highest gene expression levels of hub genes in the automated anatomical labeling (AAL) atlas are shown.


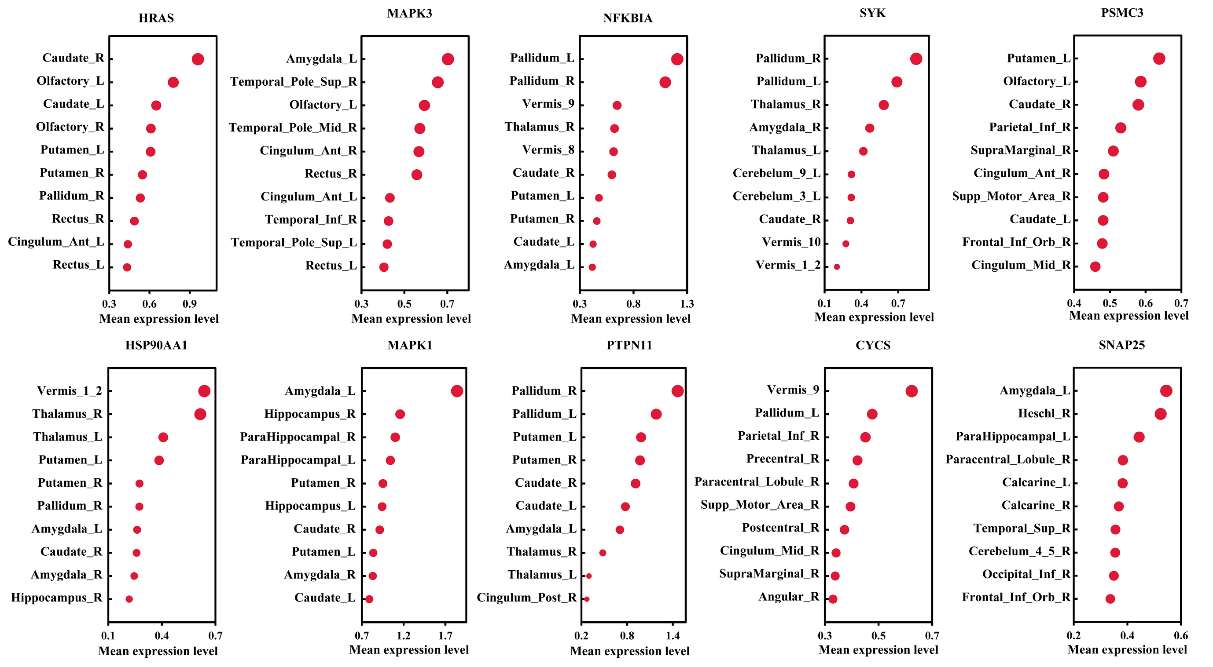

Supplement: Supplementary file 1 — Figure S1 [file CNS-30-e14690-s001.docx]
